# Dynamic Features of the Highly Excited Vibrational States of the HOCl Non-Integrable System Based on the Dynamic Potential and Lyapunov Exponent Approaches

**DOI:** 10.3390/molecules22010101

**Published:** 2017-01-07

**Authors:** Aixing Wang, Chao Fang, Yibao Liu

**Affiliations:** 1 Jiangxi Key Laboratory for Mass Spectrometry and Instrumentation, East China University of Technology, Nanchang 330013, China; xingxing_fz@sina.com (A.W.); liuyb01@mails.tsinghua.edu.cn (Y.L.); 2 School of Science, East China University of Technology, Nanchang 330013, China; 3 Institute of Nuclear and New Energy Technology, Tsinghua University, Beijing 100084, China; 4 Key Laboratory of Advanced Reactor Engineering and Safety of Ministry of Education, Beijing 100084, China; 5 Collaborative Innovation Center of Advanced Nuclear Energy Technology, Beijing 100084, China

**Keywords:** HOCl, non-integrable, highly excited vibrational state, dynamic potential, Lyapunov exponent

## Abstract

In this article the dynamic features of the highly excited vibrational states of the hypochlorous acid (HOCl) non-integrable system are studied using the dynamic potential and Lyapunov exponent approaches. On the condition that the 3:1 resonance between the H–O stretching and H–O–Cl bending modes accompany the 2:1 Fermi resonance between the O–Cl stretching and H–O–Cl bending modes, it is found that the dynamic potentials of the highly excited vibrational states vary regularly with different Polyad numbers (*P* numbers). As the *P* number increases, the dynamic potentials of the H–O stretching mode remain the same, but those of the H–O–Cl bending mode gradually become complex. In order to investigate the chaotic and stable features of the highly excited vibrational states of the HOCl non-integrable system, the Lyapunov exponents of different energy levels lying in the dynamic potentials of the H–O–Cl bending mode (*P* = 4 and 5) are calculated. It is shown that the Lyapunov exponents of the energy levels staying in the junction of Morse potential and inverse Morse potential are relative large, which indicates the degrees of chaos for these energy levels is relatively high, but the stabilities of the corresponding states are good. These results could be interpreted as the intramolecular vibrational relaxation (IVR) acting strongly via the HOCl bending motion and causing energy transfers among different modes. Based on the previous studies, these conclusions seem to be generally valid to some extent for non-integrable triatomic molecules.

## 1. Introduction

The chaotic vibrational dynamics of the hypochlorous acid (HOCl) non-integrable system has aroused widespread interest [1,2,3]. The interaction among the three vibrational modes of HOCl (H–O stretching mode, O–Cl stretching mode and H–O–Cl bending mode) makes the molecular system non-integrable and its dynamic features are more complicated than those of the integrable system [4,5,6]. Numerous studies using quantum ab-initio calculations and classical bifurcation analysis on the nonlinear coupling of the O–Cl stretching and H–O–Cl bending modes were undertaken, and in recent years, semi-classical methods represented by the dynamic potential method have brought a new approach to this issue [7,8,9,10,11,12,13]. In previous work, the HOCl integrable system, which is governed by the 2:1 Fermi resonance between the O–Cl stretching mode and the H–O–Cl bending mode, was investigated and it was found that the dynamic potential approach could illustrate the dynamic features visually using geometrical patterns [10]. Specifically, this approach shows its superiority in that it is very simple and shows a clear picture, both methodologically and physically.

There is 3:1 resonance between the H–O stretching mode and the H–O–Cl bending mode accompanying the 2:1 Fermi resonance between the O–Cl stretching mode and the H–O–Cl bending mode [2,3,4] in the HOCl non-integrable system, which indicates the dynamic features of this system are complicated and interesting. However, the physical connotations behind this are unclear so far because the traditional ab-initio calculations for this complex nonlinear system are extremely burdensome and it is hard to get a clear picture. As a new way, the dynamic features of the highly excited vibrational states of the HOCl non-integrable system are studied using the dynamic potential approach in the paper. With the semi-classical Hamiltonian, the dynamic potentials under different Polyad numbers are obtained and a comparative analysis is done. In addition, the chaotic and stable features of the highly excited vibrational states of the HOCl non-integrable system are studied by calculating the corresponding Lyapunov exponents.

## 2. Semi-Classical Hamiltonian of HOCl Non-Integrable System and Corresponding Dynamic Characterizing Methods

### 2.1. Semi-Classical Hamiltonian of HOCl Non-Integrable System

The dissociation energy of HOCl (X^1^*A*’, electronic ground state) is 19,289.6 cm^−1^ (experimental value) [1] and the highly excited energy region studied in this work is 7 × 10^3^ cm^−1^–1.8 × 10^4^ cm^−1^, where the experimental data of energy levels are plentiful (350 levels) and the dynamic features are attractive [2,3,4]. Considering 3:1 resonance and 2:1 resonance, the Hamiltonian could be expressed as with second quantization form:

(1) $$\begin{array}{l} H=\omega_{1}(n_{1}+\frac{1}{2})+\omega_{2}(n_{2}+\frac{1}{2})+\omega_{3}(n_{3}+\frac{1}{2})\\ +X_{11}{\left(n_{1}+\frac{1}{2}\right)}^{2}+X_{22}{\left(n_{2}+\frac{1}{2}\right)}^{2}+X_{33}{\left(n_{3}+\frac{1}{2}\right)}^{2}\\ +X_{12}(n_{1}+\frac{1}{2})(n_{2}+\frac{1}{2})+X_{23}(n_{2}+\frac{1}{2})(n_{3}+\frac{1}{2})\\ +y_{111}{\left(n_{1}+\frac{1}{2}\right)}^{3}+y_{333}{\left(n_{3}+\frac{1}{2}\right)}^{3}+y_{122}(n_{1}+\frac{1}{2}){\left(n_{2}+\frac{1}{2}\right)}^{2}\\ +y_{133}(n_{1}+\frac{1}{2}){\left(n_{3}+\frac{1}{2}\right)}^{2}+y_{223}{\left(n_{2}+\frac{1}{2}\right)}^{2}(n_{3}+\frac{1}{2})+y_{233}(n_{2}+\frac{1}{2}){\left(n_{3}+\frac{1}{2}\right)}^{2}\\ +z_{2222}{\left(n_{2}+\frac{1}{2}\right)}^{4}+z_{3333}{\left(n_{3}+\frac{1}{2}\right)}^{4}+z_{1122}{\left(n_{1}+\frac{1}{2}\right)}^{2}{\left(n_{2}+\frac{1}{2}\right)}^{2}+z_{1222}(n_{1}+\frac{1}{2}){\left(n_{2}+\frac{1}{2}\right)}^{3}\\ +z_{2333}(n_{2}+\frac{1}{2}){\left(n_{3}+\frac{1}{2}\right)}^{3}+z_{1233}(n_{1}+\frac{1}{2})(n_{2}+\frac{1}{2}){\left(n_{3}+\frac{1}{2}\right)}^{2}\\ +z_{22222}{\left(n_{2}+\frac{1}{2}\right)}^{5}+z_{22333}{\left(n_{2}+\frac{1}{2}\right)}^{2}{\left(n_{3}+\frac{1}{2}\right)}^{3}\\ +[k+k_{2}n_{2}+k_{3}(n_{3}+\frac{3}{2})+k_{22}n_{2}^{2}+k_{23}n_{2}(n_{3}+\frac{3}{2})+k_{33}{\left(n_{3}+\frac{3}{2}\right)}^{2}](a_{2}^{+}a_{3}^{2}+a_{3}^{+2}a_{2})\\ +\mathrm{KKK}(a_{1}a_{2}^{+3}+a_{2}^{3}a_{1}^{+}) \end{array}$$

Subscripts 1, 2, and 3 of the Hamiltonian respectively correspond to the H–O stretching vibrational mode, the H–O–Cl bending vibrational mode and the O–Cl stretching vibrational mode. *a*^+^ and *a* indicate the increase or decrease of quantum number of different vibrational modes. *n* is quantum number of vibrational modes and for convenience, hereinafter we use *n_i_* (*i* = 1, 2, 3) to denote the corresponding vibrational modes, whose corresponding position coordinates are marked by *q_i_* and the momentum coordinates are marked by *p_i_*, respectively. ω is the simple harmonic oscillation coefficient and *X*, *y*, *z*, *k*, KKK denote the nonlinear coupling coefficients among three different modes. For this Hamiltonian, a matrix can be constructed by the basis states |*n*_1_|*n*_2_|*n*_3_ > and the eigenvalues can be obtained for fitting the level energies to elucidate the Hamiltonian coefficients. This process has been done [1] and the results are given here in Table 1.

The energy level fitting rms error is 5.29 cm^−1^ and the maximum error is 27.08 cm^−1^ [1]. In particular, the coupling coefficients between *n*_2_ and *n*_3_ of the non-integrable Hamiltonian are similar to the ones of the integrable Hamiltonian, which means the effect of the H–O stretching mode on the 2:1 Fermi resonance between *n*_2_ and *n*_3_ is weak.

Because of the coupling between the *n*_1_ and *n*_2_ modes, and the coupling between the *n*_2_ and *n*_3_ modes in the molecular system, according to the theory of conserved quantity, the additional conserved quantity of the HOCl non-integrable Hamiltonian can be expressed by *n*_1_ + *n*_2_/3 + *n*_3_/6, which is called the Polyad number (*P* number) [7,8,9,10,11,12]. There is a specified dynamic potential corresponding to each *P* number and it is easy to find that there are (*P* + 1)(*P* + 2)/2 energy levels for every specified *P* [8]. On the other hand, the corresponding coset space in the Lie group of the vibration Hamiltonian of HOCl is SU(3)/U(2) and through general method of semi-classification of Hamiltonians [14], the semi-classical expression of Equation (1) could be obtained as the following three representations:

(2) H(q1,p1,q3,p3,P)=ω1(p12+q122+12)+ω2(3P−3(p12+q12)2−p32+q324+12)+ω3(p32+q322+12)+X11(p12+q122+12)2+X22(3P−3(p12+q12)2−p32+q324+12)2+X33(p32+q322+12)2+X12(p12+q122+12)(3P−3(p12+q12)2−p32+q324+12)+X23(3P−3(p12+q12)2−p32+q324+12)(p32+q322+12)+y111(p12+q122+12)3+y333(p32+q322+12)3+y122(p12+q122+12)(3P−3(p12+q12)2−p32+q324+12)2+y133(p12+q122+12)(p32+q322+12)2+y223(3P−3(p12+q12)2−p32+q324+12)2(p32+q322+12)+y233(3P−3(p12+q12)2−p32+q324+12)(p32+q322+12)2+z2222(3P−3(p12+q12)2−p32+q324+12)4+z3333(p32+q322+12)4+z1122(p12+q122+12)2(P−3(p12+q12)2−p32+q324+12)2+z1222(p12+q122+12)(3P−3(p12+q12)2−p32+q324+12)3+z2333(3P−3(p12+q12)2−p32+q324+12)(p32+q322+12)3+z1233(p12+q122+12)(3P−3(p12+q12)2−p32+q324+12)(p32+q322+12)2+z22222(3P−3(p12+q12)2−p32+q324+12)5+z22333(3P−3(p12+q12)2−p32+q324+12)2(p32+q322+12)3+[k+k2(3P−3(p12+q12)2−p32+q324)+k3(p32+q322+32)+k22(3P−3(p12+q12)2−p32+q324)2+k23(3P−3(p12+q12)2−p32+q324)(p32+q322+32)+k33(p32+q322+32)2]•3P−3(p12+q12)2−p32+q324(q32−p32)+KKK2q1(3P−3(p12+q12)2−p32+q324)32

(3) $$\begin{array}{l} H(q_{2},p_{2},q_{3},p_{3},P)=\omega_{1}(P-\frac{p_{2}^{2}+q_{2}^{2}}{6}-\frac{p_{3}^{2}+q_{3}^{2}}{12}+\frac{1}{2})+\omega_{2}(\frac{p_{2}^{2}+q_{2}^{2}}{2}+\frac{1}{2})+\omega_{3}(\frac{p_{3}^{2}+q_{3}^{2}}{2}+\frac{1}{2})\\ +X_{11}{\left(P-\frac{p_{2}^{2}+q_{2}^{2}}{6}-\frac{p_{3}^{2}+q_{3}^{2}}{12}+\frac{1}{2}\right)}^{2}+X_{22}{\left(\frac{p_{2}^{2}+q_{2}^{2}}{2}+\frac{1}{2}\right)}^{2}+X_{33}{\left(\frac{p_{3}^{2}+q_{3}^{2}}{2}+\frac{1}{2}\right)}^{2}\\ +X_{12}(P-\frac{p_{2}^{2}+q_{2}^{2}}{6}-\frac{p_{3}^{2}+q_{3}^{2}}{12}+\frac{1}{2})(\frac{p_{2}^{2}+q_{2}^{2}}{2}+\frac{1}{2})+X_{23}(\frac{p_{2}^{2}+q_{2}^{2}}{2}+\frac{1}{2})(\frac{p_{3}^{2}+q_{3}^{2}}{2}+\frac{1}{2})\\ +y_{111}{\left(P-\frac{p_{2}^{2}+q_{2}^{2}}{6}-\frac{p_{3}^{2}+q_{3}^{2}}{12}+\frac{1}{2}\right)}^{3}+y_{333}{\left(\frac{p_{3}^{2}+q_{3}^{2}}{2}+\frac{1}{2}\right)}^{3}\\ +y_{122}(P-\frac{p_{2}^{2}+q_{2}^{2}}{6}-\frac{p_{3}^{2}+q_{3}^{2}}{12}+\frac{1}{2}){\left(\frac{p_{2}^{2}+q_{2}^{2}}{2}+\frac{1}{2}\right)}^{2}+y_{133}(P-\frac{p_{2}^{2}+q_{2}^{2}}{6}-\frac{p_{3}^{2}+q_{3}^{2}}{12}+\frac{1}{2})\\ {\left(\frac{p_{3}^{2}+q_{3}^{2}}{2}+\frac{1}{2}\right)}^{2}+y_{223}{\left(\frac{p_{2}^{2}+q_{2}^{2}}{2}+\frac{1}{2}\right)}^{2}(\frac{p_{3}^{2}+q_{3}^{2}}{2}+\frac{1}{2})+y_{233}(\frac{p_{2}^{2}+q_{2}^{2}}{2}+\frac{1}{2}){\left(\frac{p_{3}^{2}+q_{3}^{2}}{2}+\frac{1}{2}\right)}^{2}\\ +z_{2222}{\left(\frac{p_{2}^{2}+q_{2}^{2}}{2}+\frac{1}{2}\right)}^{4}+z_{3333}{\left(\frac{p_{3}^{2}+q_{3}^{2}}{2}+\frac{1}{2}\right)}^{4}+z_{1122}{\left(P-\frac{p_{2}^{2}+q_{2}^{2}}{6}-\frac{p_{3}^{2}+q_{3}^{2}}{12}+\frac{1}{2}\right)}^{2}\\ {\left(\frac{p_{2}^{2}+q_{2}^{2}}{2}+\frac{1}{2}\right)}^{2}+z_{1222}(P-\frac{p_{2}^{2}+q_{2}^{2}}{6}-\frac{p_{3}^{2}+q_{3}^{2}}{12}+\frac{1}{2}){\left(\frac{p_{2}^{2}+q_{2}^{2}}{2}+\frac{1}{2}\right)}^{3}+z_{2333}(\frac{p_{2}^{2}+q_{2}^{2}}{2}+\frac{1}{2})\\ {\left(\frac{p_{3}^{2}+q_{3}^{2}}{2}+\frac{1}{2}\right)}^{3}+z_{1233}(P-\frac{p_{2}^{2}+q_{2}^{2}}{6}-\frac{p_{3}^{2}+q_{3}^{2}}{12}+\frac{1}{2})(\frac{p_{2}^{2}+q_{2}^{2}}{2}+\frac{1}{2}){\left(\frac{p_{3}^{2}+q_{3}^{2}}{2}+\frac{1}{2}\right)}^{2}\\ +z_{22222}{\left(\frac{p_{2}^{2}+q_{2}^{2}}{2}+\frac{1}{2}\right)}^{5}+z_{22333}{\left(\frac{p_{2}^{2}+q_{2}^{2}}{2}+\frac{1}{2}\right)}^{2}{\left(\frac{p_{3}^{2}+q_{3}^{2}}{2}+\frac{1}{2}\right)}^{3}+[k+k_{2}(\frac{p_{2}^{2}+q_{2}^{2}}{2})\\ +k_{3}(\frac{p_{3}^{2}+q_{3}^{2}}{2}+\frac{3}{2})+k_{22}{\left(\frac{p_{2}^{2}+q_{2}^{2}}{2}\right)}^{2}+k_{23}(\frac{p_{2}^{2}+q_{2}^{2}}{2})(\frac{p_{3}^{2}+q_{3}^{2}}{2}+\frac{3}{2})\\ +k_{33}{\left(\frac{p_{3}^{2}+q_{3}^{2}}{2}+\frac{3}{2}\right)}^{2}](q_{2}q_{3}^{2}-q_{2}p_{3}^{2}+2q_{3}p_{2}p_{3})/\sqrt{2}\\ +\mathrm{KKK}\sqrt{\frac{P}{2}-\frac{p_{2}^{2}+q_{2}^{2}}{12}-\frac{p_{3}^{2}+q_{3}^{2}}{24}}(q_{2}^{3}-3q_{2}p_{2}^{2}) \end{array}$$

(4) $$\begin{array}{l} H(q_{1},p_{1},q_{2},p_{2},P)=\omega_{1}(\frac{p_{1}^{2}+q_{1}^{2}}{2}+\frac{1}{2})+\omega_{2}(\frac{p_{2}^{2}+q_{2}^{2}}{2}+\frac{1}{2})\\ +\omega_{3}(6P-3(p_{1}^{2}+q_{1}^{2})-(p_{2}^{2}+q_{2}^{2})+\frac{1}{2})+X_{11}{\left(\frac{p_{1}^{2}+q_{1}^{2}}{2}+\frac{1}{2}\right)}^{2}+X_{22}{\left(\frac{p_{2}^{2}+q_{2}^{2}}{2}+\frac{1}{2}\right)}^{2}\\ +X_{33}{\left(6P-3(p_{1}^{2}+q_{1}^{2})-(p_{2}^{2}+q_{2}^{2})+\frac{1}{2}\right)}^{2}+X_{12}(\frac{p_{1}^{2}+q_{1}^{2}}{2}+\frac{1}{2})(\frac{p_{2}^{2}+q_{2}^{2}}{2}+\frac{1}{2})\\ +X_{23}(\frac{p_{2}^{2}+q_{2}^{2}}{2}+\frac{1}{2})(6P-3(p_{1}^{2}+q_{1}^{2})-(p_{2}^{2}+q_{2}^{2})+\frac{1}{2})+y_{111}{\left(\frac{p_{1}^{2}+q_{1}^{2}}{2}+\frac{1}{2}\right)}^{3}\\ +y_{333}{\left(6N-3(p_{1}^{2}+q_{1}^{2})-(p_{2}^{2}+q_{2}^{2})+\frac{1}{2}\right)}^{3}+y_{122}(\frac{p_{1}^{2}+q_{1}^{2}}{2}+\frac{1}{2}){\left(\frac{p_{2}^{2}+q_{2}^{2}}{2}+\frac{1}{2}\right)}^{2}\\ +y_{133}(\frac{p_{1}^{2}+q_{1}^{2}}{2}+\frac{1}{2}){\left(6P-3(p_{1}^{2}+q_{1}^{2})-(p_{2}^{2}+q_{2}^{2})+\frac{1}{2}\right)}^{2}+y_{223}{\left(\frac{p_{2}^{2}+q_{2}^{2}}{2}+\frac{1}{2}\right)}^{2}\\ (6P-3(p_{1}^{2}+q_{1}^{2})-(p_{2}^{2}+q_{2}^{2})+\frac{1}{2})+y_{233}(\frac{p_{2}^{2}+q_{2}^{2}}{2}+\frac{1}{2}){\left(6P-3(p_{1}^{2}+q_{1}^{2})-(p_{2}^{2}+q_{2}^{2})+\frac{1}{2}\right)}^{2}\\ +z_{2222}{\left(\frac{p_{2}^{2}+q_{2}^{2}}{2}+\frac{1}{2}\right)}^{4}+z_{3333}{\left(6P-3(p_{1}^{2}+q_{1}^{2})-(p_{2}^{2}+q_{2}^{2})+\frac{1}{2}\right)}^{4}+z_{1122}{\left(\frac{p_{1}^{2}+q_{1}^{2}}{2}+\frac{1}{2}\right)}^{2}\\ {\left(\frac{p_{2}^{2}+q_{2}^{2}}{2}+\frac{1}{2}\right)}^{2}+z_{1222}(\frac{p_{1}^{2}+q_{1}^{2}}{2}+\frac{1}{2}){\left(\frac{p_{2}^{2}+q_{2}^{2}}{2}+\frac{1}{2}\right)}^{3}+z_{2333}(\frac{p_{2}^{2}+q_{2}^{2}}{2}+\frac{1}{2})(6P-3(p_{1}^{2}+q_{1}^{2})\\ -(p_{2}^{2}+q_{2}^{2})+\frac{1}{2}{)}^{3}+z_{1233}(\frac{p_{1}^{2}+q_{1}^{2}}{2}+\frac{1}{2})(\frac{p_{2}^{2}+q_{2}^{2}}{2}+\frac{1}{2}){\left(6P-3(p_{1}^{2}+q_{1}^{2})-(p_{2}^{2}+q_{2}^{2})+\frac{1}{2}\right)}^{2}\\ +z_{22222}{\left(\frac{p_{2}^{2}+q_{2}^{2}}{2}+\frac{1}{2}\right)}^{5}+z_{22333}{\left(\frac{p_{2}^{2}+q_{2}^{2}}{2}+\frac{1}{2}\right)}^{2}{\left(2P-3(p_{1}^{2}+q_{1}^{2})-(p_{2}^{2}+q_{2}^{2})+\frac{1}{2}\right)}^{3}\\ +[k+k_{2}(\frac{p_{2}^{2}+q_{2}^{2}}{2})+k_{3}(6P-3(p_{1}^{2}+q_{1}^{2})-(p_{2}^{2}+q_{2}^{2})+\frac{3}{2})+k_{22}{\left(\frac{p_{2}^{2}+q_{2}^{2}}{2}\right)}^{2}+k_{23}(\frac{p_{2}^{2}+q_{2}^{2}}{2})\\ \left(6P-3(p_{1}^{2}+q_{1}^{2})-(p_{2}^{2}+q_{2}^{2})+\frac{3}{2})+k_{33}{\left(6P-3(p_{1}^{2}+q_{1}^{2})-(p_{2}^{2}+q_{2}^{2})+\frac{3}{2}\right)}^{2}\right]\\ (6P-3(p_{1}^{2}+q_{1}^{2})-(p_{2}^{2}+q_{2}^{2}))\sqrt{2}q_{2}+\mathrm{KKK}(q_{1}q_{2}^{3}-3q_{1}q_{2}p_{2}^{2}+3p_{1}p_{2}q_{2}^{2}-p_{1}p_{2}^{3})/2 \end{array}$$

In this transformation, the equations $n_{j}=\left(q_{j}^{2}+p_{j}^{2}\right)/2$, $n_{k}=\left[P-\sum \alpha_{j}(q_{j}^{2}+p_{j}^{2})/2\right]/\alpha_{j}$ are used. Here, *n_j_* denote any two different modes of *n*_1_, *n*_2_, *n*_3_ and *n_k_* denotes a residual one. *α_j_* is the front coefficient of *n_j_* in the equation *P* = 1·*n*_1_ + 1/3·*n*_2_ + 1/6·*n*_3_ (*j* = 1,2,3). Considering the semi-classical method is mainly applicable to highly excited states of molecular systems, in the following study, the semi-classical Hamiltonian would be used to analyze the dynamic potential of *P* = 2, 3, 4, 5.

### 2.2. Dynamic Potential Methods for Non-Integrable System

The dynamic potential of *H*(*p*,*q*,*P*) is the effective environment in which the *q* coordinate stays for each *P* in a certain molecule. This is achieved by calculating the maximal and minimal energies by varying *p* for each *q* under the condition that the corresponding conserved quantum number is non-negative. The dynamic potential composed of these maximal and minimal energies as a function of *q* is represented by a closed curve in which the quantal levels are enclosed and also defines the *q* region for each level it encloses [7,8,9]. From a dynamic potential perspective, it is easy to understand the dynamic information of each highly excited vibrational state in spectroscopy visually, such as the localization, dissociation, isomerization, chaotic and even the energy transfers between different modes in a molecule, which is significant for the study of highly excited vibrational dynamics [9,10,11,12,13]. For a better exploration of the HOCl vibrational dynamics, we need to calculate the dynamic potentials of HOCl. With the Hamiltonian *H*(*q_i_*,*p_i_*,*q_j_*,*p_j_*,*P*) (*i* = 1, 2, 3; *j* = 1, 2, 3; *i* ≠ *j*) of HOCl, for a certain *P* number, the process is done by varying (*p_i_*,*p_j_*) to obtain maximum energy *E_+_* and minimum energy *E_−_* for each (*q_i_*,*q_j_*) under the constraint $\alpha_{i}\left(q_{i}^{2}+p_{i}^{2}\right)+\alpha_{j}\left(q_{j}^{2}+p_{j}^{2}\right)\leq 2P$, which guarantees *n_k_* is positive. Thus *E_+_*(*q_i_*,*q_j_*) and *E_−_*(*q_i_*,*q_j_*) determine the dynamic potential for a certain *P* number and the energy levels share the same *P* number are all contained in a closed surface shaped by *E*_+_ and *E*_−_. Furthermore, the points in the dynamic potential corresponding to ∂H/∂q=0 are called fixed points in the dynamic space, which governs the various quantal environments in which the vibrational states lie [10,11,12,13].

For convenience, the closed two-dimensional dynamic potential curve of *E_+_*(*q_i_* = 0,*q_j_*) and *E_−_*(*q_i_* = 0,*q_j_*) is considered in the following discussion [8]. In particular, it is found that the dynamic potentials can be obtained by three different coset representations of the Hamiltonian of a non-integrable triatomic molecule system and in the following section, it will be demonstrated that though the dynamic potentials obtained with different coset representations of the Hamiltonian are not strictly the same, the shapes of the dynamic potentials and corresponding fixed points are almost similar for a certain coordinates-momentum representation. 

### 2.3. Lyapunov Exponent—Chaotic Index of Non-Integrable Systems

The Lyapunov exponent, which shows the rate of change of the separation divergence of two neighboring trajectories in the phase coset space, can be used to characterize the degree of chaos of a non-integrable system [14,15,16]. In the calculation, we may choose a point together with its neighboring point in the coset space and then follow their corresponding trajectories determined by Hamilton’s equations of motion. For a certain energy level, the equation *H*(*q_i_*,*p_i_*,*q_j_*,*p_j_*,*P*) = *E_s_*, (*i* = 1, 2, 3; *j* = 1, 2, 3, *i* ≠ *j*; *Es* is the value of a certain energy level) would be resolved, then the solution of the system would be worked out by canonical equation as follows:

(5) $$\begin{array}{l} dq_{i}/dt=\partial H/\partial p_{j}\\ dp_{j}/dt=-\partial H/\partial q_{i} \end{array}$$

Giving another point that is $\Delta x_{0}$ away from the initial point, the distance of these two points’ trajectory is $\Delta x_{T}$ at time T and the functional relationship between $\Delta x_{0}$ and $\Delta x_{T}$ is:

(6) $$\Delta x_{T}=e^{\lambda T}\Delta x_{0}$$

The parameter λ is the Lyapunov exponent and if λ > 0, the system is chaotic and more chaotic the higher the value of λ [17]. λ can be resolved concretely in the following way: firstly, setting an initial point *x*(0) and another point *x*(0)’ which fulfill the equation $\left|x\left(0\right)-x\left(0\right)’\right|=d_{0}$ on the same curved energy surface. After a time interval *T*_1_ = τ, these two points will become *x*(τ) and *x*(τ)’ which fulfill the equation $\left|x\left(\tau \right)-x\left(\tau \right)’\right|=d_{1}$ with the evolution of the Hamilton equations of the system. Then on the segment line of points *x*(τ) and *x*(τ)’, a point *x*(τ)” can be obtained which fulfills the equation $\left|x\left(\tau \right)-x\left(\tau \right)”\right|=d_{0}$. Taking *x*(τ)’ and *x*(τ)” as initial values, after an interval *T*_2_ = 2τ, *x*(τ)’ and *x*(τ)” would become *x*(2τ)’ and *x*(2τ)” with the evolution of Hamilton’s equations, which fulfill the equation $\left|x\left(2\tau \right)-x\left(2\tau \right)”\right|=d_{2}$. Repeating these steps, a series of *d_i_*(1,2,3…) could be obtained and average Lyapunov exponent λ is given by the expression:

(7) $$\lambda =\underset{n\to \infty }{\lim}\frac{1}{n\tau }[\sum_{i=1}^{n}\ln(d_{i}/d_{0})]$$

The details of this calculation can be found in [15,16,17]. In the calculation, the convergence of the exponent is checked and the maximal average Lyapunov exponent is considered [8]. In the following discussion, “Lyapunov exponent” represents “maximal average Lyapunov exponent” for short.

## 3. Dynamic Features of Highly Excited Vibrational States in HOCl Non-Integrabel System

In a previous study, it is found that the dynamic features could be clarified visually by the dynamic potentials and Lyapunov exponents [8]. In this section, the varying patterns of the dynamic potentials under different *P* numbers and their dynamic connotative meanings in a non-integrable HOCl system will be studied firstly. Secondly, the chaotic features of highly excited vibrational states will be shown through a comparative analysis between the Lyapunov exponent and the dynamic potential under some certain *P* value conditions.

### 3.1. Dynamic Potentials and Their Different Coordinates Representation Features for a Typical P Number

**Figure 1:**
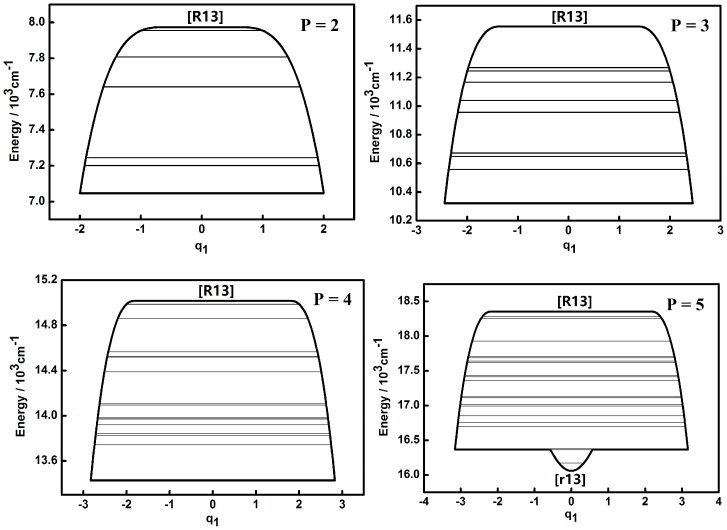
Dynamic potentials of the non-integrable HOCl system (obtained with the *H*(*q*_1_,*p*_1_,*q*_3_,*p*_3_,*P*), *q*_1_ coordinate) with *P* = 2, 3, 4, 5, and the energy levels included in the dynamic potential are shown by the lines.

**Figure 2:**
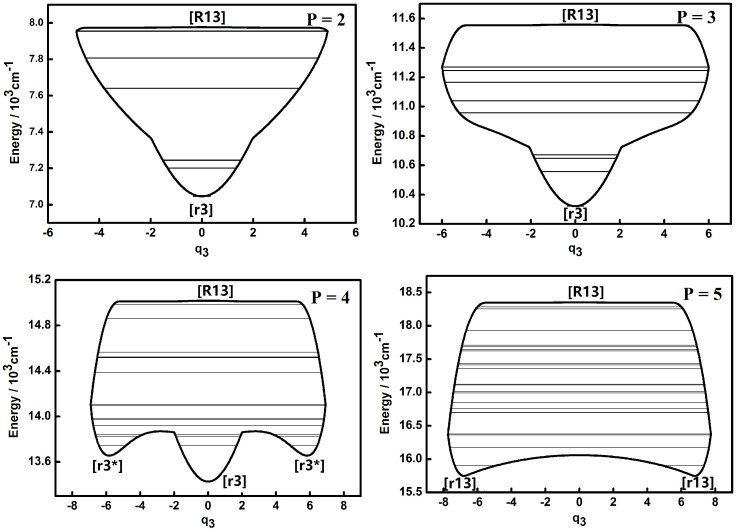
Dynamic potentials of the non-integrable HOCl system (obtained with *H*(*q*_1_,*p*_1_,*q*_3_,*p*_3_,*P*), *q*_3_, coordinate) with *P* = 2, 3, 4, 5, and the energy levels included in the dynamic potential are shown by the lines.

The dynamic potentials of the non-integrable HOCl system obtained with *H*(*q*_1_,*p*_1_,*q*_3_,*p*_3_,*P*) are shown in Figure 1 and Figure 2. 

A fixed point in the inverse Morse type potential (Morse type potential) of the H–O, O–Cl stretching modes is denoted as [R] ([r]) and the one of the bending mode is denoted as [B] ([b]). A “*” superscript added to [R] ([r]) or [B] ([b]) is for the secondary appearance of [R] ([r]) or [B] ([b]) type fixed point in a dynamic potential. An additional subscript 1, 2, 3 for a fixed point is a reminder of the appearance in the dynamic potential of coordinate *q*_1_,*q*_2_,*q*_3_. This will be used in the following discussion. It is shown that the dynamic potentials of *q*_1_ under different *P* numbers are all very simple and the patterns of all the dynamic potentials are simple inverse Morse types as *P* = 2, 3, 4, which indicates that the stability of higher energy levels is superior to that of lower ones [7,8,9,10]. A fixed point appears at the bottom of the pattern in the case of *P* = 5, which indicates that the vibration corresponding to the lowest energy level is localized and its stability is better compared with other states which share a common *P* number. The dynamic features contained in the dynamic potentials of *q*_3_ are relatively more complicated. When *P* = 2, the shape of the dynamic potential is a Morse type one and the stability of lower energy levels is superior to that of higher ones. As the value of *P* increases, there are two new stable fixed points ([r3*]) at the bottom of the dynamic potential pattern, which means that a localized vibrational mode in a limited range appears. When *P* is up to 5, the original stable fixed point ([r3]) at the bottom of pattern would disappear and the dynamic potential pattern tends to be simple, while the two fixed points ([r13]) at the bottom of the dynamic potential pattern stay the same, which indicates that these two points are insensitive to the increase of the vibrational energy of the whole system.

**Figure 3:**
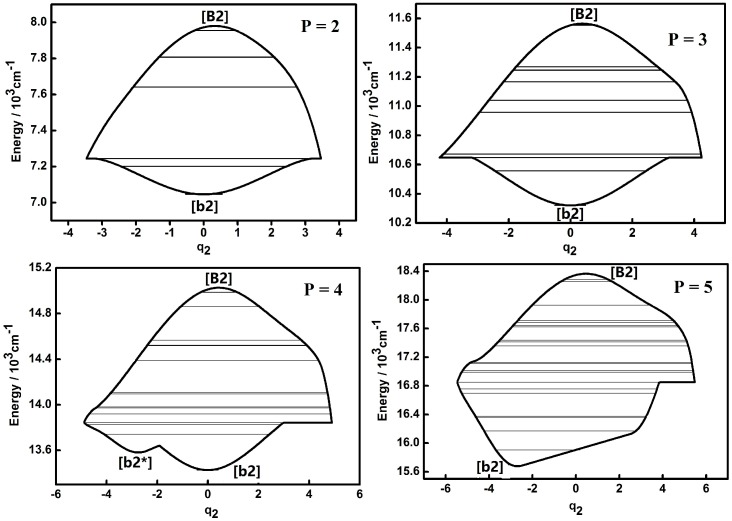
Dynamic potentials of the non-integrable HOCl system (obtained with *H*(*q*_2_,*p*_2_,*q*_3_,*p*_3_,*P*), *q*_2_ coordinate) with *P* = 2, 3, 4, 5, and the energy levels included in the dynamic potential are shown by the lines.

**Figure 4:**
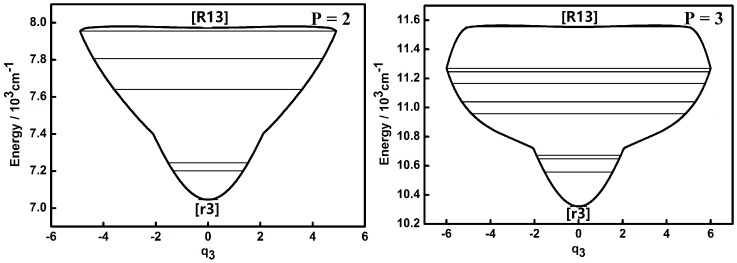

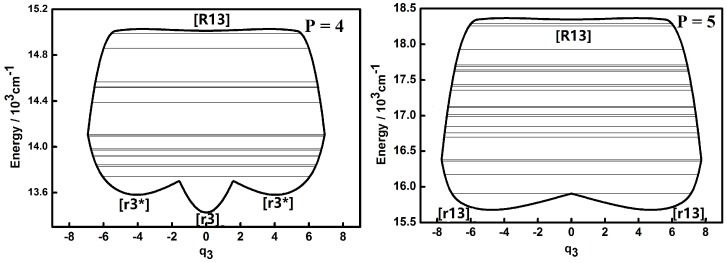
Dynamic potentials of the non-integrable HOCl system (obtained with *H*(*q*_2_,*p*_2_,*q*_3_,*p*_3_,*P*), *q*_3_ coordinate) with *P* = 2, 3, 4, 5, and the energy levels included in the dynamic potential are shown by the lines.

The dynamic potentials of the non-integrable HOCl system obtained with *H*(*q*_2_,*p*_2_,*q*_3_,*p*_3_,*P*) are shown in Figure 3 and Figure 4. In these figures it is found that the dynamic potentials of *q*_2_ are similar under different *P* values, but the fixed points are different. As *P* is increasing, a new fixed point ([b2*]) appears at the bottom of the pattern when *P* is up to 4. When *P* = 5, one of fixed point in the dynamic potential when *P* = 4 in the center area disappears, while another one remains. What should be noticed is that the dynamic potentials of *q*_3_ obtained with *H*(*q*_2_,*p*_2_,*q*_3_,*p*_3_,*P*) are similar with the ones obtained with *H*(*q*_1_,*p*_1_,*q*_3_,*p*_3_,*P*), especially the fixed points which are almost the same. This indicates that the dynamic potentials for a certain *P* and a certain coordinate representation, corresponding fixed points would not change. This conclusion shows that in the future work for triatomic non-integrable systems, repeating the analysis of dynamic potential of a specified coordinate representation from two different types of Hamiltonian is not necessary, which is similar with the case of the integrable system.

**Figure 5:**
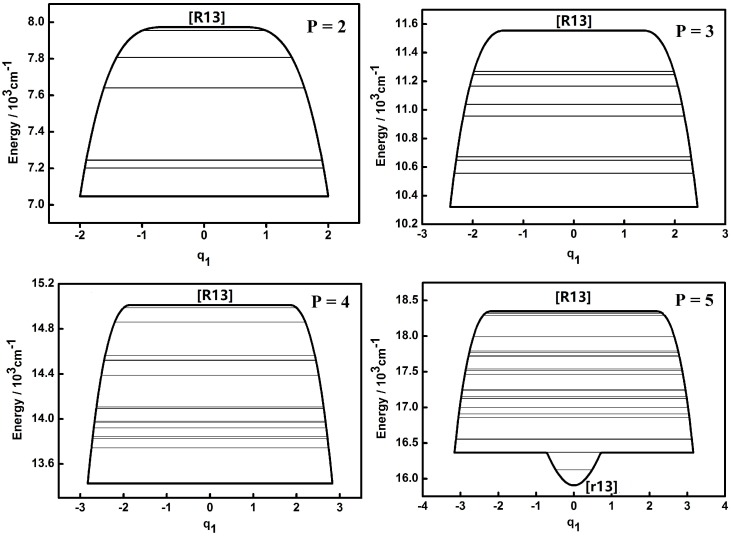
Dynamic potentials of the non-integrable HOCl system (obtained with *H*(*q*_1_,*p*_1_,*q*_2_,*p*_2_,*P*), *q*_1_ coordinate) with *P* = 2, 3, 4, 5, and the energy levels included in the dynamic potential are shown by the lines.

**Figure 6:**
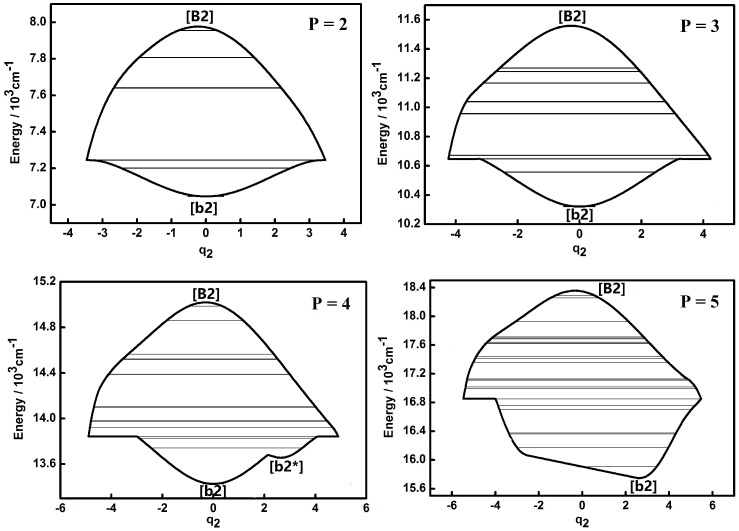
Dynamic potentials of the non-integrable HOCl system (obtained with *H*(*q*_1_,*p*_1_,*q*_2_,*p*_2_,*P*), *q*_2_ coordinate) with *P* = 2, 3, 4, 5, and the energy levels included in the dynamic potential are shown by the lines.

The dynamic potentials of non-integrable HOCl system obtained with *H*(*q*_1_,*p*_1_,*q*_2_,*p*_2_,*P*) are shown in Figure 5 and Figure 6.

In Figure 5 and Figure 6, it is seen that dynamic potentials of *q*_1_ and *q*_2_ obtained with *H*(*q*_1_,*p*_1_,*q*_2_,*p*_2_,*P*) are just the mirror inversion in the *q* coordinate compared the ones obtained with *H*(*q*_1_,*p*_1_,*q*_3_,*p*_3_,*P*) *H*(*q*_2_,*p*_2_,*q*_3_,*p*_3_,*P*), respectively. Because the dynamic potentials of *q*_1_ are is bilaterally symmetric, they stay the same with the representation of *H*(*q*_1_,*p*_1_,*q*_2_,*p*_2_,*P*) or *H*(*q*_1_,*p*_1_,*q*_3_,*p*_3_,*P*). On the other hand, the observed clipping direction of the bending mode in the *q*_1_ coordinate is opposite to the result for the *q*_3_ coordinate and it is easy to know that the mirror inversion of dynamic potentials is due to the reversal of the vibrational reference frame. Just like the results mentioned in the previous section, the fixed points remain the same in the dynamic potentials of *q*_1_ and *q*_2_ obtained with *H*(*q*_1_,*p*_1_,*q*_2_,*p*_2_,*P*) compared with the results obtained with *H*(*q*_1_,*p*_1_,*q*_3_,*p*_3_,*P*) and *H*(*q*_2_,*p*_2_,*q*_3_,*p*_3_,*P*), respectively, which indicates the dynamic features obtained before are independent of the different coset representations of the Hamiltonians.

Compared to the integrable HOCl system [10,11], the dynamic potential of the non-integrable HOCl system is simpler. Though only the 2:1 resonance between the O–Cl stretching mode and the H–O–Cl bending mode is considered in the integrable system, the number of fixed points in the dynamic potentials under different *P* values are significantly different [11]. On the other hand, the 3:1 Fermi resonance of the H–O stretching mode and the H–O–Cl bending mode makes the dynamic features of the non-integrable HOCl system simple, which indicates that the number of coupling modes considered in a molecular system does not directly affect the complexity of the dynamic features of the system. From Figure 1, Figure 2, Figure 3, Figure 4, Figure 5 and Figure 6, it is easy to get an overview of the dynamic potentials’ evolution of the HOCl non-integrable system under different *P* numbers and the dynamic features of some certain states (such as localizations). With the increase of *P*, the dynamic potentials of the H–O stretching mode don’t change greatly, but the dynamic potentials of the H–O–Cl bending mode become more complicated.

### 3.2. Lyapunov Exponents and Chaotic Features of Highly Excited Vibrational States under Certain P number

**Figure 7:**
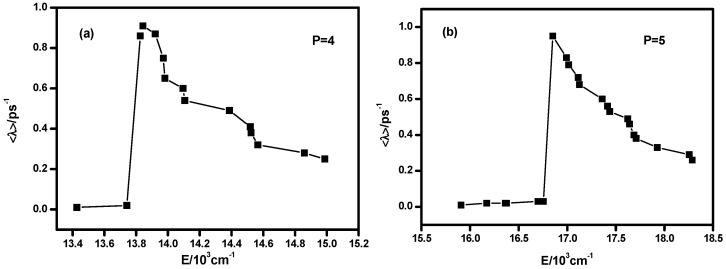
Lyapunov exponent of every highly excited vibrational state when (**a**) *P* = 4 and (**b**) *P* = 5.

In previous work, it is shown that the Lyapunov exponent is the degree of chaos of a highly excited vibrational state and with a larger Lyapunov exponent, the stability of highly excited vibrational energy levela is better because the energy could be transferred among different modes and avoids being accumulated in a certain mode, which would prevent the dissociation or isomerization [8,18]. In order to verify whether this conclusion is applicable to the non-integrable HOCl system, the Lyapunov exponent of every energy level is calculated when *P* = 4 (15 energy levels included) and *P* = 5 (21 energy levels included). The results are shown in Figure 7 (the Lyapunov exponents obtained with *H*(*q*_1_,*p*_1_,*q*_2_,*p*_2_,*P*), *H*(*q*_1_,*p*_1_,*q*_3_,*p*_3_,*P*) and *H*(*q*_2_,*p*_2_,*q*_3_,*p*_3_,*P*) are almost same, so here we use the results of *H*(*q*_1_,*p*_1_,*q*_2_,*p*_2_,*P*)).

As shown in Figure 7 and comparison with the dynamic potentials of *q*_2_ (Figure 3 and Figure 6), it is found that the Lyapunov exponents of the energy levels in the Morse dynamic potential are small both in case (a) and (b), which indicates that the vibrational modes of these energy levels are regular, simple and energy transferring through intramolecular vibrational relaxation (IVR) between the three different modes (H–O stretching, H–O–Cl bending and O–Cl stretch) is weak [18,19]. On the other hand, the Lyapunov exponents of the energy levels in the inverse Morse dynamic potential are large, which indicates that vibrational modes of these energy levels are much more chaotic and the IVR effect is much more obvious than the one in the Morse dynamic potential. Furthermore, the energy level corresponding the maximum value of Lyapunov exponent is the one that lies in the junction of the Morse and inverse Morse dynamic potential, where the motion range of vibration reaches the maximum and it could be elucidated that the IVR effect is strong. The above results demonstrate that the occurrence of energy transfers among the three modes and the appearance of large nonzero Lyapunov exponents are well correlated. This is logical in the sense that the bending motion often plays a pumping role in the energy exchange between the two stretching motions in three-body dynamics. This mediation by the bending motion is also favorable for the IVR, thereby enhancing the degree of dynamic chaos, which leads to a larger Lyapunov exponent. The energy flow mediated by the bending motion between the H–O and O–Cl stretches facilitates the relaxation of the energy accumulated in the H–C bond and makes the system stable instead of facilitating dissociation, which agrees with the previous work [8].

In Figure 3 and Figure 5 of [1], the features of these states are shown by the probability densities of the vibrational wave functions obtained by ab-initio potential energy surface (EPS) calculations. Our result indicates that the three lowest energy states when *P* = 4 and the six lowest energy states when *P* = 5 possess mainly HOCl bending motion and the IVR is strong. This is consistent with the results in [1]. From the comprehensive analysis of the Lyapunov exponent and dynamic potentials, the distinction and differentiation of different states are easy to find, which could also be demonstrated by the probability densities of wave functions. However, the level of difficulty of the two different ways are incomparable. On the other hand, the ab-initio EPS could not show the exact wave function of 3-mode coupling of the non-integrable HOCl system in [1] because of the heavy computational burden, but in our work the results are very easy to obtain.

## 4. Conclusions and Remarks

The above studies suggest that considering the 3:1 resonance between the H–O stretching and H–O–Cl bending modes adding the 2:1 Fermi resonance between the O–Cl stretching and H–O–Cl bending modes, the dynamic potentials of these three modes change regularly with different Polyad numbers. With the increase of Polyad number, the dynamic potentials of the H–O stretching mode don’t change greatly, but the dynamic potentials of the H–O–Cl bending mode become more complicated. Particularly, when considering the 3:1 resonance, the dynamic features of the highly excited vibrational states are greatly different from the ones of the integrable HOCl system only governed by 2:1 Fermi resonance, which means that the 3:1 resonance is not negligible. With the analysis of the Lyapunov exponents of different energy levels when *P* = 4 and 5, it is found that the vibrational modes of low energy levels contained in the Morse type dynamic potential are rigid and the corresponding Lyapunov exponents are relative small. However, the vibrational modes of high energy levels contained in the inverse Morse type dynamic potential are chaotic and their Lyapunov exponents are relative large. The energy level corresponding to the maximum Lyapunov exponent is the one that lies in the junction of the Morse and inverse Morse potentials, which means the range of corresponding vibrational motions is the largest. It is also found that the Lyapunov exponents are well correlated with the mediation by the bending motion in IVR and the bending motion often plays a pumping role in the energy exchange between the two stretching motions in three-body dynamics. Considering the similar results of HCO and DCO [8,18], it seems that these conclusions are generally valid to some extent for triatomic molecules in general, which enables us to understand non-integrable dynamics of triatomic molecules simply from their geometrical pattern without repeated and complex ab-initio calculation elaboration. 

## Figures and Tables

**Table 1:** The coefficients of the vibration Hamiltonian of the HOCl non-integrable system.

Parameter	Values (cm^−1^)	Parameter	Values (cm^−1^)
ω_1_	3777.067	*z*_2222_	−0.04117
ω_2_	1258.914	*z*_3333_	−0.00171
ω_3_	753.834	*z*_1122_	−0.15070
*X*_11_	−80.277	*z*_1222_	0.13189
*X*_12_	−19.985	*z*_2333_	−0.01229
*X*_22_	−3.204	*z*_1233_	0.02381
*X*_23_	−10.637	*z*_22222_	0.00151
*X*_33_	−7.123	*z*_22333_	−0.00066
*y*_111_	−0.3619	*k*	0
*y*_333_	0.0825	*k*_2_	−0.76017
*y*_122_	−1.9534	*k*_3_	−0.24939
*y*_133_	−0.0532	*k*_22_	−0.01158
*y*_223_	−0.0802	*k*_23_	0.04075
*y*_233_	−0.2503	*k*_33_	0.00583
KKK	0.19520		
